# ‘The mean mummy way’ – experiences of parents instilling eye drops to their young children as described in online forums and blogs

**DOI:** 10.1186/s12887-020-02410-4

**Published:** 2020-11-10

**Authors:** Gloria C. Law, Alpaslan Bülbül, Christina J. Jones, Helen Smith

**Affiliations:** 1 Lee Kong Chian School of Medicine, Nanyang Technological University Singapore, Singapore, 308232 Singapore; 2 Brighton and Sussex Medical School, Brighton, UK; 3 School of Psychology, University of Surrey, Guildford, UK

**Keywords:** Primary health care, Pediatrics, Eye drop administration, Parents, Young children, Qualitative study, Social media

## Abstract

**Background:**

Adults often have difficulty instilling eye drops in their own eyes, but little has been documented about the difficulties experienced by parents when administering eye drops to their young children, where the challenges of instillation are accentuated by their inability to cooperate. This qualitative study explores parents’ experiences of administering eye drops to their children as described in online forum discussions and blog posts.

**Methods:**

This was an exploratory study using qualitative methods. We thematically analysed the written exchanges between parents participating in online forum discussions and blog posts about the administration of eye drops to their young children.

**Results:**

We found 64 forum discussion threads and 4 blog posts, representing 464 unique contributors expressing their experiences of eye drop administration to young children. Three major themes were identified – administration challenges, administration methods and role of health care professionals. Besides describing their children’s distress, parents discussed their own discomfort and anxiety when administering eye drops. Parents used a variety of techniques to facilitate adherence with medication, including restraining the child, role-play, reassurance, distraction, or reward. The ideas exchanged about eye drop administration occasionally included reiteration of professional advice, but were dominated by parents’ own ideas/suggestions; interestingly health care professionals were considered diagnosticians and prescribers, rather than sources of practical advice on administration.

**Conclusions:**

Parents struggling to deliver eye drops to their young children may seek advice on how to administer treatment from parental on-line discussion forums. The distress experienced by the young child and their parents is a powerful reminder to clinicians that procedures common and routine in health care may be challenging to parents. The advice given to parents needs to go beyond the instillation of the eye drops, and include advice on child restraint, distraction techniques and allaying distress. Forewarned of the potential difficulties and provided with coping strategies parents can employ when the child resists, could alleviate their own and their child’s distress.

## Background

Adults frequently have difficulty instilling eye drops in their own eyes [1], and this can be observed even in patients who self-report no difficulties in administration of drops [2]. Examples of poor techniques include touching of the eye or ocular adnexa with the bottle, poor handling or squeezing of the eye drop container, difficulty aiming at the eyes, dispensing multiple drops and needing multiple attempts before successful instillation [3, 4]. The challenges of administering eye drops to infants and young children is further exacerbated by their poorer understanding and ability to cooperate. In ophthalmological inpatient and outpatient settings, where eyedrops are administered by health care professionals, as many as a quarter of pre-school children have been observed to be distressed and uncooperative [5–7]. It has been observed that the younger the child the more likely they are to be distressed by eye drops administration [8]. To date little research has focused on the difficulties experienced by parents when administering eye drops to their young children in their own homes.

The purpose of the study was to explore and analyse parents’ experiences administering eye drops to young children using online forum discussions and blog posts. Online forums are an alternative to face-to-face interviews or focus groups as sources of qualitative data. Online forums provide a comfortable way to discuss personal health issues and are effective in providing emotional and informational support for participants [9]. This asynchronous mode of discussion is convenient and accessible and although there have been concerns about the credibility and accuracy of information shared online these appear to be infrequent [10] and largely overshadowed by the benefits reported, such as social support [11]. Another rich source of data on people’s experiences are weblogs, or blogs as they are commonly referred to. Blogs consist of a series of updated, chronologically ordered posts, usually written by a single author. They are publicly available and provide substantial amounts of data which can be collected at a low-cost [12].

## Methods

### Data collection

We first identified websites where parents discussed the care of children by conducting searches using three web search engines (Google, Yahoo and Bing). This resulted in 25 potential UK websites in English (single discussion fora threads, single blog posts and larger active parenting websites). To identify relevant threads each site was then searched for the following keywords ‘how to eye drops toddler’, ‘tips eye drops toddler’, ‘tricks eye drops toddler’, ‘child refusing eye drops’, ‘advice eye drops toddler’, ‘help eye drops toddler’, ‘best way eye drops toddler’. In addition to using the term *toddler*, we also used the keywords *baby, son, daughter, dd (dear daughter), ds (dear son) and child* to reflect commonly used words and internet abbreviations. The threads were downloaded and extracted between 7 and 12.1.15. (See *Data Extraction Summary,* Table 1). Each thread was identified with two descriptors: (1) the number of the forum (F) or blog (B), and (2) the initials of the sender’s ‘name’, so ‘F2, C’ indicating the quote came from forum number 2, and was contributed by a parent with pseudo initial ‘C’. For the purpose of this study, parents were defined as caregivers of young children including fathers, mothers or guardians, and young children are defined as infants and toddlers. The time and date of postings were recorded if available.

**Table 1:** Data Extraction Summary

Online discussion forums (F) or Blogs (B)	Number of forum or blog threads	No. of unique names identified	Range of dates of posts	Extraction date
F1	2	9	7 years ago	12-01-2015
		8	5 years ago	
F2	10	10	07-06-2014 to 08-06-2014	07-01-2015
		5	03-04-2014	
		6	04-12-2012	
		5	14-11-2011 to 15-11-2011	
		9	30-11-2014 to 02-12-2014	
		7	22-01-2013 to 23-01-2013	
		5	15-02-2012 to 16-02-2012	
		6	14-09-2011	
		6	02-11-2011	
		9	23-01-2013 to 25-01-2013	
F3	7	9	31-12-2012	07-01-2015
		5	07-06-2010	
		8	07-07-2014	
		8	30-03-2013	
		5	11-01-2012 to 15-01-2012	
		8	26-02-2011	
		7	22-03-2010 to 22-04-2010	
F4	2	5	22-03-2009	12-01-2015
		12	11-01-2015 to 13-01-2015	
F5	1	11	24-07-2010	09-01-2015
F6	1	7	No dates recorded	12-01-2015
B1	1	1	No dates recorded	12-01-2015
F7	1	9	No dates recorded	12-01-2015
B2	1	1	No dates recorded	12-01-2015
F8	9	4	08-05-2007	07-01-2015
		10	29-07-2011 to 30-07-2011	
		7	03-09-2012 to 05-09-2012	
		4	30-09-2006 to 01-10-2006	
		5	09-09-2005 to 11-09-2005	
		6	06-01-2005 to 09-01-2005	
		12	19-09-2006 to 20-09-2006	
		4	10-08-2009 to 11-08-2009	
		9	26-09-2008 to 27-09-2008	
F9	1	4	11-05-2007	12-01-2015
F10	1	7	08-10-2012	12-01-2015
F11	1	8	No dates recorded	12-01-2015
F12	1	2	19-04-2011	12-01-2015
F13	1	4	26-03-2009	12-01-2015
F14	6	6	23-04-2007 to 25-04-2007	12-01-2015 & 15-01-2015
		18	23-10-2008 to 24-10-2008	
		19	05-01-2010 to 08-01-2010	
		7	15-05-2006 to 17-05-2006	

### Data analysis

The data was analysed using Applied Thematic Analysis, a systematic and inductive approach to generate themes from textual data [13]. The downloaded transcripts from online discussion fora and blogs were read repeatedly by two researchers (AB and GL) working independently to get familiar with the data. The data was imported into the NVivo qualitative analysis computer software package for data reduction and to identify key structural topics which were then used to generate subthemes and themes from the data. These preliminary themes were presented to the other members of the research team (CJ, HS) for checking and comparison with the original data. Any disagreements were discussed to achieve a majority decision (agreement between three of the four researchers).

### Ethical considerations

As recommended by British Psychological Society guidelines on internet mediated research [14], we used only public web blogs and online forum discussions that could be accessed without signing in. To ensure anonymity and confidentiality, website addresses have not been presented. All quotes used to illustrate themes were returned to search engines to ensure their origin was not apparent, and if it was, they were paraphrased to ensure anonymity.

## Results

Fourteen (56%) of the 25 websites reviewed had discussions about parental eye drop administration to young children. Sixty-four online fora discussion threads and four blog posts, representing 464 unique sender names, were identified (Table 1). Most postings were assumed to be by mothers (female name or pseudonyms and mentioned their husband and children), with the earliest posting from 2004. Three major themes were identified; administration challenges, administration techniques and role of health care professionals (Table 2), and these are discussed in detail below.

**Table 2:** Orders of themes and descriptions of eye drop administration

Themes	Subthemes	Descriptions
1. Administration Experience	Child’s resistance to administration	Fight, scream & cry
		Shut their eyes tight
		Distress and fear
		Drops sting eyes and taste bad
	Parents’ reluctance to administer	Horrible and cruel
		Upset child
2. Administration Methods	Using force and restraint	One person pin & drop
		Two persons pin & drop
		‘Leg-over-child’s arms’ method
		Burrito-method
	“Show and tell”	Demonstration & Role-play
		Talk child through
		Praise & reward
	Minimize child’s distress & awareness	Distraction – Game or play
		Manage eye drop on closed eyes
		While sleeping
	Other management strategies	Give up eye drop administration
		Alternative ways to treatment
3. Role of healthcare professionals	Diagnosticians & prescribers	
	Guidance on eye medication administration methods	

### Administration challenges

The distress associated with the administration of eye drops was not only confined to the child, but also generated negative emotions and anxiety in parents.

#### Child’s resistance to administration

There were multiple accounts of children disliking and resisting eye drop administration, in ways that varied with the verbal and physical stages of the child’s development. Distress and fear were apparent by gestures, facial expressions and by screaming and crying.

‘Every time it's her screaming and crying murder! I bet someone walking by would think we are beating her!!! For eye drops!!!!’ (F2, C)

The physical responses were focused on escape (for example *‘wriggling’, ‘thrashing’, ‘fighting’*) and resistance, such as shutting their eyes tight to prevent the eye drops being inserted. The

‘It is such a nightmare to get her to take them she fights us and scrunches her eye so tight there is no way the drops go in.’ (F8, A)

Parents often reported how the immediate discomfort of the eye drops contributed to the child’s reluctance.

‘The drops burn and hurt, so he will instinctively fight those drops. Plus, they take a while to work which means a prolonged battle.’ (F14, AZ)

#### Parents’ reluctance to administer

Many parents also discussed their own discomfort as the administrator of eye drops; they alluded to feeling *‘a real bully’*, ‘*horrible*’ and ‘*cruel’* and were reluctant to perform this task. One parent said,

‘I’m having to employ the pin-her-down and use ‘prise eyes open’ tactic now. It's horrible, I hate having to do it!’ (F3, M)

Parents similarly expressed fear that they might hurt their child, for example:

‘We feel terrible about that and worry that she might get hurt thrashing about as she does. It is just horrible, and we still have some more days to go.’ (F15, E)

### Administration methods

Parents shared the techniques they had utilised to achieve successful administration of eye drops and to achieve a more positive experience for their child.

#### Using force and restraint

Parents described utilising force to administer eye drops. The methods of restraint employed were numerous; they described techniques that enabled administration when single-handed or when assisted by another adult. For unaccompanied administration the ‘leg-over-child’s arms’ method was frequently used:

‘With great bloody difficulty I sit on the floor ….., lay him back with my legs over his shoulders, holding his eye open with one hand and doing the drops with the other. Walah! Worst experience ever. *Bows*. I have tried every other way and it’s not as effective, so I’m stuck with the mean mummy way.’ (F5, LS)

There were other, more sophisticated, methods of restraint described, including the ‘*burrito method’* to swaddle the child so that their arms are trapped. One parent said,

‘I started wrapping him up in a bath towel like a giant baby burrito. Then, I lean over him, slide my arm under his head, and put the drops in the corner of his eye.’ (F15, AZ)

#### Show and tell

Some parents suggested gentler approaches to facilitate of eye drop administration, such as demonstrating on toys and getting the child to role-play giving medication.

‘So far the demonstration has fascinated him but doesn't stop his panicking when we put the drops in him. He even “gives” Daddy, Mommy, and Elmo the drops. Oh well, even if not all of the medicine gets in there it seems to be working.’ (F7, AY)

For a child at the age of understanding parents might try to explain why the eye drops were needed. One parent wrote,

‘I'm finding more and more that [Child] understands what I'm saying even if she can't communicate back very well and if I explain exactly what is going to happen in advance … then she usually has no problem at all.’ (F3, FY)

Other parents recommended praise or reward when the child cooperated.

‘Try giving him a lollypop as a reward and to distract him from the pain and taste. … Or let him pick a treat after he does the drops.’ (F3, JM)

#### Minimize child’s distress and awareness

Parents used distraction of many sorts (entertainment, games, and play) to minimize the child’s immediate reactions to eye drop administration. Some encouraged the child to watch TV so they would not see the eye drop bottle approaching. Others achieved compliance by putting the drops on closed eyes, as described here:

‘Did you try letting him lie down with his eyes closed? Incline his chin back a bit and put the eyedrops in the corners of his eyes...then tell him to open his eyes … It helped that they couldn't see the drop coming!’ (F14, JH)

Alternatively, parents waited until their child was asleep then lifted the eyelids and inserted the drops.

#### Other management strategies

Some parents ‘*gave up’* (their own words) on instilling the eye drops to their child because of the inherent difficulties as well as believing their child’s eye problem would ‘*clear up on its own*’ within a few days. Rather than seeing the child distress and lack of cooperation as a challenge to be overcome, some parents questioned the necessity of what they had been asked to do.

‘We gave up. [Child] had pink eye with his last ear infection and the urgent care doc prescribed eye drops that we were supposed to give every 4 hours. I called my regular doctor and asked if it was really necessary. And pink eye with drops will, on average, clear up in 3.4 days and without will clear up in 4.5 days.’ (F2, SK)

Other parents adopted alternative treatments, for example cleaning with *‘breast milk’*, or bathing the child’s eyes with *‘salt water’, ‘warm water’*, or *‘cold tea’*.

‘I expressed breastmilk into a clean bowl, dipped in cotton wool and then wiped over each eye several times a day. Cleared up in around 3 days if I remember rightly.’ (F4, FT)

Others recommended asking for ointment rather than drops, or keeping the eye drops warm to reduce stinging.

‘It did help to use warm drops. I kept the bottle in my pocket or bra, so it was body temperature.’ (F15, BT)

### Role of healthcare professionals

In these on-line discussions parents shared their own experiences, both positive and negative, of eye drop administration. The challenges were predominantly discussed in the context of parenting, with less discussion about seeking support from health care professionals and services.

#### Diagnosticians and prescribers

Parents mentioned health care professionals mostly in the context of diagnosis or prescribing treatment.

‘[Child] has mild conjunctivitis in his eyes. The doctor gave him some eye drops to use 2 times a day for 5 days.’ (F8, FF)

Only occasionally was re-consultation with a health care professional suggested to achieve a different formulation that is easier to administer and requires fewer doses:

‘Go back to the doctor, say it is impossible and ask for Fucithalmic, it is more of a cream than an eye drop, so easier to apply but also only needs to be applied a few times a day.’ (F16, A)

#### Guidance on eye medication administration methods

Rarely were health care professionals cited as a source of the advice parents were sharing on successful eye drop administration. Similarly, no reference was ever made to websites addressing eyedrop administration for the young child. When professional advice was shared it originated from a range of health care workers; doctors (general practitioners and specialists (an ophthalmologist, a paediatrician)), nurses, a paramedic and a pharmacist.

‘A recent advice from an eye specialist for giving eye drops to my toddler - if you put enough drops onto the eyelashes of a close eye, when the eye is opened, the drops will wash in …’ (F16, NE)

Unlike accounts of advice from other health professionals, the advice from nurses combined advice on administration with tips on restraint, perhaps reflecting their greater professional involvement in giving eyedrops.

‘The nurses in the NICU showed us how to do it. You have to kind of pin the baby's head so they can't move, hold the eye drops in one hand ready to use them, use your thumb and index finger of the other hand to open their eye lid and hold it open while you squeeze the eye drops into the inside corner of their eye.’ (F2, TJ)

## Discussion

### Summary of our findings

In these online discussions parents shared in detail their own unfortunate or unsuccessful experiences of administering eye drops to their young children and requested help. The parental accounts identified were very powerful, engaging, and alerted us to previously unrecognised challenges. It is apparent that young children’s dislike and resistance to the instillation of eye drops can cause significant parental distress which can reduce their willingness to continue treatment. Parents shared a variety of techniques to facilitate eye drop administration and included the use of restraint and force. The advice was framed as arising from their own experiences and often having been developed by ‘*trial and error*. As we were using textual data we were unable to explore the influence of healthcare professionals, printed or on-line information on the information shared, but whatever its origins the advice proffered was generally safe and appropriate.

### Comparison with existing literature

Parents described their dislike of resorting to force to overcome their child’s reluctance to have eyedrops. Immobilising the child to achieve compliance with therapy creates a tension between what is perceived to be in the child’s best interest and their role as a trusted care giver. In paediatric healthcare use of restraint (sometimes referred to as clinical holding [15], therapeutic holding [16] or supportive holding [17]) is not uncommon with infants and children up to school age [18, 19]. Whilst holding children for clinical interventions may be uncontested in clinical practice nurses, like parents, have also expressed concerns and a preference to avoid restraint so as not to harm their caring relationship with the child [20, 21]. Restraint can impact on children in many ways; there is the frequently observed generation of fear, anger, confusion and emotional stress [22, 23], but restraint may also cause or exacerbate pain [24], inflict injury and cause speech disturbance if used excessively [25]. The longer-term psychological sequelae include phobias and difficulty establishing relationships with health care professional [26]. However, from the descriptors of techniques shared online by parents they appear to be advising methods that are commonly accepted as safe, although we acknowledge that we were unable to assess the severity of the force used [27].

### Implications for clinical practice

Having highlighted the difficulties experienced by some parents we need to consider possible solutions. The use of eye drop instillation aids may improve parental dexterity when administering eye drops to their children [28] and replacing liquid drops with ointment may also help. There is a recognised need for clinicians to describe and demonstrate eye drop administration techniques at the time of prescribing and pharmacists to reinforce these messages when dispensing the drops or ointment. Our findings highlight a need for this advice to be extended to include tips on managing a young child’s reluctance to stay still and for parents to manage their own anxieties and distress. There are resources already available for training health care staff in restraint, for example “Evidence-based holding of children for clinical procedures” [28] and there are guidelines and information to help healthcare professionals to educate caregivers to instil eye drops to babies and young children [29, 30]. In a brief informal review of currently available patient information leaflets and relevant websites we observed that they are generally strong on administration of medications into the eye, but less informative about dealing with an uncooperative recipient. Advising parents how to deal with their young child’s reticence to having eye drops would be better addressed proactively as there may be difficulty changing the course of action once the child has developed an uncooperative pattern of behaviour.

### Strengths and limitations

The use of online discussion sites as a data source for research is an emerging technique [31]. Using data generated by a group that was not been convened for research purposes, sometimes referred to as ‘naturally occurring’ [32] or ‘non-reactive’ [14] data, has advantages; because the researcher is not leading data collection and is ‘invisible’ to the participants there is no opportunity to influence the discussion or encourage socially desirable responses. Text presentation was sometimes informal, with errors of literacy and spelling, but the messages were always clear. Whilst some of the data is from several years ago, it remains relevant as children’s dislike of eye drops persists as reflected in recent publications (e.g. [33]). The major limitations of using online discussions as a data source are that the lack of demographic details of contributors, and researchers having no opportunity to ask follow-up questions. Online discussion excludes those people without internet access, and this may differentially exclude poorer families. In contrast, the use of the internet may enable the voices of some commonly excluded people (for example, those with disabling conditions, parents of young children, or those living in rural settings) to participate in research. Unlike verbal interactions, online forums enable people to respond at their convenience instead of waiting their turn, providing an opportunity for more reserved participants to contribute [34]. Even with such methodological weaknesses these parental online discussions have generated unique data that we hope will in turn impact on clinical practice.

## Conclusion

This study highlights how some parents struggle to deliver eye drops to their young children and seek advice on how to administer treatment from parental on-line discussion forums. In some instances, the amount of distress experienced by the young child and their parent appears significant; being aware of these difficult scenarios is a powerful reminder to clinicians that procedures which are common and routine in health care may represent unchartered territory for parents. The advice given to parents needs to go beyond the instillation of the eye drops, and include advice on child restraint, distraction techniques and allaying distress. If parents are forewarned of the difficulties and given coping strategies they can employ when the child objects or resists, this will decrease both their own and their child’s distress.

## Data Availability

The datasets used and/or analysed during the current study are available from the corresponding author on reasonable request.

## References

[1] Al-Busaidi A, Samek DA, Kasner O (2016). Eye drop administration in patients attending and not attending a glaucoma education center. Oman J Ophthalmol.

[2] Schwartz GF, Hollander DA, Williams JM (2013). Evaluation of eye drop administration technique in patients with glaucoma or ocular hypertension. Curr Med Res Opin.

[3] Adamson E, Kendall G (2016). Difficulty in eye drop administration for people with rheumatoid arthritis. Br J Occup Ther.

[4] Sayner R, Carpenter DM, Robin AL, Blalock SJ, Muir KW, Vitko M (2016). How glaucoma patient characteristics, self-efficacy and patient–provider communication are associated with eye drop technique. Int J Pharm Pract.

[5] Shah P, Jacks AS, Adams GGW (1997). Paediatric Cycloplegia: a new approach. Eye..

[6] Sujuan JL, Handa S, Perera C, Chia A (2015). The psychological impact of eyedrop administration in children. J APPOS.

[7] Lim J, Chia A, Saffari SE, Handa S (2019). Factors affecting pupil reactivity after cycloplegia in Asian children. Asia-Pacific Acad Ophthalmol (Phila).

[8] Hirji N, Jones S, Thompson G (2012). The causes of distress in Paediatric outpatients receiving dilating drops. Open J Ophthalmol.

[9] Im E-O, Chee W (2006). An online forum as a qualitative research method: practical issues. Nurs Res.

[10] Cole J, Watkins C, Kleine D (2016). Health advice from internet discussion forums: how bad is dangerous?. J Med Internet Res.

[11] Hajli MN, Sims J, Featherman M, Love PE (2015). Credibility of information in online communities. J Strateg Mark.

[12] Hookway N (2008). Entering the blogosphere’: some strategies for using blogs in social research. Qual Res.

[13] Guest G, MacQueen KM, Namey EE (2012). Applied thematic analysis.

[14] British Psychological Society (2013). Ethics guidelines for internet-mediated research.

[15] Labrenos K, McArthur E (2003). Introducing a clinical holding policy. Paediatr Nurs.

[16] Royal College of Nursing (2019). Restrictive physical interventions and the clinical holding of children and young people: guidance for nursing staff.

[17] Jeffery K (2010). Supportive holding or restraint: terminology and practice. Paediatr Nurs.

[18] Bray L, Carter B, Ford K, Dickinson A, Water T, Blake L (2018). Holding children for procedures: an international survey of health professionals. J Child Health Care.

[19] Kirwan L, Coyne I (2017). Use of restraint with hospitalized children: a survey of nurses’ perceptions of practices. J Child Health Care.

[20] Svendsen E, Bjørk I (2014). Experienced nurses’ use of non-pharmacological approaches comprise more than relief from pain. J Pediatr Nurs.

[21] Svendsen E, Pedersen R, Moen A, Bjørk I (2017). Exploring perspectives on restraint during medical procedures in paediatric care: a qualitative interview study with nurses and physicians. Int J Qual Stud Health Well Being.

[22] Bray L, Snodin J, Carter B (2015). Holding and restraining children for clinical procedures within an acute care setting: an ethical consideration of the evidence. Nurs Inq.

[23] Brenner M (2013). A need to protect: parents’ experiences of the practice of restricting a child for a clinical procedure in hospital. Issues Compr Paediatr Nurs.

[24] McMurtry CM, Ridell RP, Taddio A, Racine N, Asmundson GJ, Noel M (2015). Far from “just a poke”: common painful needle procedures and the development of needle fear. Clin J Pain.

[25] Forrester K, Fox-Young S, Huff N (2002). “Holding the child down” for treatment in paediatric haematology: the ethical, legal and practice implications. J Law Med.

[26] Brenner M, Parahoo K, Taggart L (2007). Child restraint: addressing the distress. J Clin Nurs.

[27] Crellin D, Babl FE, Sullivan TP, Cheng J, O'Sullivan R, Hutchinson A (2011). Procedural restraint use in preverbal and early-verbal children. Pediatr Emerg Care.

[28] Page A, Warren A, Vanes N (2017). Developing a website to demonstrate clinical holding techniques. Nurs Child Young People.

[29] Davies I, Williams AM, Muir KW (2017). Aids for eye drop administration. Surv Ophthalmol.

[30] Mason I, Stevens S (2010). Instilling eye drops and ointment in a baby or young child. Commun Eye Health.

[31] Smith H, Bulbul A, Jones CJ (2017). Can online discussion sites generate quality data for research purposes?. Front Public Health.

[32] Potter J, Hepburn A (2005). Qualitative interviews in psychology: problems and possibilities. Qual Res Psychol.

[33] Pilon F, Veen H, Kef S, van Genderen MM (2020). The impact of an eyedrop booklet on distress in children when receiving eye drops. Strabismus..

[34] Robin AL. Instilling drops: adherence is a more complex issue than it at first appears. Galucoma Today. 2010:44–5.

